# Implementation and outcome of minimally invasive pancreatoduodenectomy in Europe: a registry-based retrospective study – a critical appraisal of the first 3 years of the E-MIPS registry

**DOI:** 10.1097/JS9.0000000000001121

**Published:** 2024-01-24

**Authors:** Anouk M.L.H. Emmen, Nine de Graaf, I.E. Khatkov, O.R. Busch, S. Dokmak, Ugo Boggi, Bas Groot Koerkamp, Giovanni Ferrari, I.Q. Molenaar, Olivier Saint-Marc, Marco Ramera, Daan J. Lips, J.S.D. Mieog, Misha D.P. Luyer, Tobias Keck, Mathieu D’Hondt, F.R. Souche, Bjørn Edwin, Thilo Hackert, M.S.L. Liem, Abdallah Iben-Khayat, H.C. van Santvoort, Michele Mazzola, Roeland F. de Wilde, E.F. Kauffmann, Beatrice Aussilhou, Sebastiaan Festen, R. Izrailov, P. Tyutyunnik, M.G. Besselink, Mohammad Abu Hilal

**Affiliations:** a Department of General Surgery, Istituto Ospedaliero Fondazione Poliambulanza, Brescia; b Division of General and Transplant Surgery, University of Pisa, Pisa; c Department of Oncological and Minimally Invasive Surgery, ASST Grande Ospedale Metropolitano Niguarda, Milan, Italy; d Department of Surgery, Moscow Clinical Scientific Center, Moscow, Russia; e Department of HPB Surgery and Liver Transplantation, Beaujon Hospital, University Paris Cité, Clichy; f Service de Chirurgie Digestive, Endocrinienne et Thoracique, Centre Hospitalier Universitaire Orleans, Orleans; g Département de Chirurgie Digestive (A), Mini-invasive et Oncologique, Hôpital Saint-Eloi, Montpellier, France; h Department of Surgery, Amsterdam UMC, University of Amsterdam; i Cancer Center Amsterdam; j Department of Surgery, Onze Lieve Vrouwe Gasthuis, Amsterdam; k Department of Surgery, Medisch Spectrum Twente, Enschede; l Department of Surgery, Leiden University Medical Center, Leiden; m Department of Surgery, Catharina Hospital, Eindhoven; n Department of Surgery, Erasmus MC Cancer Institute, Rotterdam; o Department of Surgery, Regional Academic Cancer Center Utrecht, St. Antonius Hospital and University Medical Center, Utrecht, The Netherlands; p Department of Surgery, AZ Groeninge Hospital, Kortrijk, Belgium; q The Intervention Centre and Department of HPB Surgery, Oslo University Hospital and Institute for Clinical Medicine, Oslo, Norway; r Department of General, Visceral, and Thoracic Surgery, University Hospital Hamburg-Eppendorf, Hamburg; s Clinic for Surgery, University of Schleswig-Holstein Campus Lübeck, Lübeck, Germany

**Keywords:** laparoscopy, minimally invasive surgery, pancreatic surgery, pancreatoduodenectomy, registry, robot-assisted

## Abstract

**Background:**

International multicenter audit-based studies focusing on the outcome of minimally invasive pancreatoduodenectomy (MIPD) are lacking. The European Registry for Minimally Invasive Pancreatic Surgery (E-MIPS) is the E-AHPBA endorsed registry aimed to monitor and safeguard the introduction of MIPD in Europe.

**Materials and Methods:**

A planned analysis of outcomes among consecutive patients after MIPD from 45 centers in 14 European countries in the E-MIPS registry (2019–2021). The main outcomes of interest were major morbidity (Clavien–Dindo grade ≥3) and 30-day/in-hospital mortality.

**Results:**

Overall, 1336 patients after MIPD were included [835 robot-assisted (R-MIPD) and 501 laparoscopic MIPD (L-MIPD)]. Overall, 20 centers performed R-MIPD, 15 centers L-MIPD, and 10 centers both. Between 2019 and 2021, the rate of centers performing L-MIPD decreased from 46.9 to 25%, whereas for R-MIPD this increased from 46.9 to 65.6%. Overall, the rate of major morbidity was 41.2%, 30-day/in-hospital mortality 4.5%, conversion rate 9.7%, postoperative pancreatic fistula grade B/C 22.7%, and postpancreatectomy hemorrhage grade B/C 10.8%. Median length of hospital stay was 12 days (IQR 8–21). A lower rate of major morbidity, postoperative pancreatic fistula grade B/C, postpancreatectomy hemorrhage grade B/C, delayed gastric emptying grade B/C, percutaneous drainage, and readmission was found after L-MIPD. The number of centers meeting the Miami Guidelines volume cut-off of ≥20 MIPDs annually increased from 9 (28.1%) in 2019 to 12 (37.5%) in 2021 (*P*=0.424). Rates of conversion (7.4 vs. 14.8% *P*<0.001) and reoperation (8.9 vs. 15.1% *P*<0.001) were lower in centers, which fulfilled the Miami volume cut-off.

**Conclusion:**

During the first 3 years of the pan-European E-MIPS registry, morbidity and mortality rates after MIPD were acceptable. A shift is ongoing from L-MIPD to R-MIPD. Variations in outcomes between the two minimally invasive approaches and the impact of the volume cut-off should be further evaluated over a longer time period.

## Introduction

Highlights

- This international multicenter audit-based study analyzed 1336 patients after minimally invasive pancreatoduodenectomy (MIPD) (835 robot-assisted and 501 laparoscopic) from 45 centers in 14 countries in the European Registry for Minimally Invasive Pancreatic Surgery (E-MIPS; 2019–2021).
- The rate of major morbidity after MIPD was 41.2%, 30-day/in-hospital mortality rate 4.5%, and conversion 9.7%.
- An ongoing shift from laparoscopic to robot-assisted pancreatoduodenectomy was observed with an increasing number of centers meeting the Miami Guidelines volume cut-off (≥20 MIPD annually).

Over the past decade, minimally invasive pancreatoduodenectomy (MIPD) has gained popularity, aiming to reduce surgical trauma, enhance postoperative recovery, and reduce morbidity^1–4^. Several studies from expert centers have reported promising results on MIPD in selected patients, leading to an increased implementation of MIPD internationally^5,6^. However, concerns exist about the safety of MIPD during the implementation phase, especially since MIPD is associated with a strong volume-outcome relationship and outcomes can vary widely between hospitals and surgeons^7^.

In 2019, the Miami International Evidence-Based Guidelines on Minimally Invasive Pancreas Resection were established. These guidelines emphasized the need for structured training coupled with participation in national and international registries to facilitate outcome assessment for the safe and controlled implementation of MIPD^8^. Subsequently, in 2022, the Brescia internationally validated European Guidelines meeting on Minimally Invasive Pancreatic Surgery (EGUMIPS) confirmed that those performing MIPD should participate in a registry or have a prospective database to monitor and report on outcomes^9^. So far, multicenter studies reporting on international MIPD outcomes from international registries are lacking.

In 2019, the European Consortium on Minimally Invasive Pancreatic Surgery (E-MIPS) registry was established, designed to monitor and safeguard the introduction of robot-assisted and laparoscopic MIPD (R-MIPD, L-MIPD) in low-volume and high-volume centers across Europe^10^. The aim of this study is to report the results of the first three years of the E-MIPS registry on patients scheduled to undergo MIPD, and to provide an overview of the use and outcomes of MIPD across Europe.

## Methods

### Study design and participants

This is a planned analysis of the prospectively maintained E-MIPS registry (2019–2021). Overall, 72 centers in 21 European countries are participating in E-MIPS. Of these, 45 centers (45/72, 62.5%) in 14 countries (14/21, 67%) perform MIPD. Data from all patients who underwent R-MIPD and L-MIPD between 1 January 2019 and 31 December 2021 were included. Excluded were other pancreatic procedures, hybrid approaches (reconstruction phase open) and procedures with insufficient baseline data or missing primary outcome data. All cases were registered anonymously by the participating centers via an online secured CASTOR system (CASTOR, CIWIT B.V.), comprising all parameters of interest, including definitions. At each site, a local study coordinator was appointed for data collection of baseline characteristics, pathological parameters, and short-term outcomes (until discharge and/or up until 30 days postdischarge). Per year, three audit visits were performed by the E-MIPS registry coordinators in randomly selected centers to perform a data quality check and to ensure data validity. In May 2023, all centers received a questionnaire with center-specific questions regarding experience in the use of R-MIPD and L-MIPD over time and the timing of potential shift from L-MIPD to R-MIPD. This study has been reported in line with the strengthening the reporting of cohort, cross-sectional, and case–control studies in surgery (STROCSS) criteria^11^.

### Endpoints

Baseline patient characteristics included sex, age, American Society of Anaesthesiologists (ASA) classification, BMI, kg/m^2^, comorbidity, previous abdominal surgery, administration of (neo) adjuvant chemo-therapy or radiotherapy (if indicated), vascular involvement on preoperative imaging, additional organ involvement and presence of a dilated pancreatic duct greater than 5 mm.

Intraoperative variables included operative time (min), estimated blood loss (ml), conversion, and drain placement. Postoperative outcomes (30-day/in-hospital) included major morbidity [Clavien–Dindo (CD) grade ≥3]^12^, mortality, postoperative pancreatic fistula (POPF)^13^, delayed gastric emptying^14^, postpancreatectomy hemorrhage (PPH)^15^, surgical site infection^16^, reinterventions (radiologic, endoscopic, and surgical), length of hospital stay (days) and readmissions (<30 days). For ISGPS defined complications, only those clinically relevant (grade B/C) were included. Oncological variables included histopathological tumor type, total number of retrieved lymph nodes, number of positive lymph nodes in the specimen, resection margin status^17^, and tumor size in the resected specimen. Postoperative and follow-up data was limited to primary hospital admission and/or 30 days after surgery.

### Ethics

Ethical approval was waived due to the observational nature of the study.

### Subgroup analyses

Subgroup analysis was performed for surgical approach (R-MIPD vs. L-MIPD) and center volume, based on the Miami guideline cut-off of ≥20 MIPDs/year (low-volume vs. high-volume centers)^8^.

### Statistical analysis

Data analyses were performed by two study coordinators (AE, NdG) using IBM SPSS Statistics for Windows version 28.0 (IBM Corp.) and R’s programming environment (R Foundation for Statistical Computing). Student’s *t*, Mann–Whitney *U*, χ^2^, or Fisher’s exact tests were used for comparisons where appropriate. Categorical data is presented as proportions, continuous data is presented as either mean and SD, or as median and interquartile range, as appropriate. A *P*-value <0.050 was used to indicate statistical significance.

## Results

Between 1 January 2019 and 1 January 2022, data from 1336 patients after MIPD from 45 centers in 14 countries were included, comprising 835 R-MIPDs and 501 L-MIPDs.

### Center characteristics

**Figure 1:**
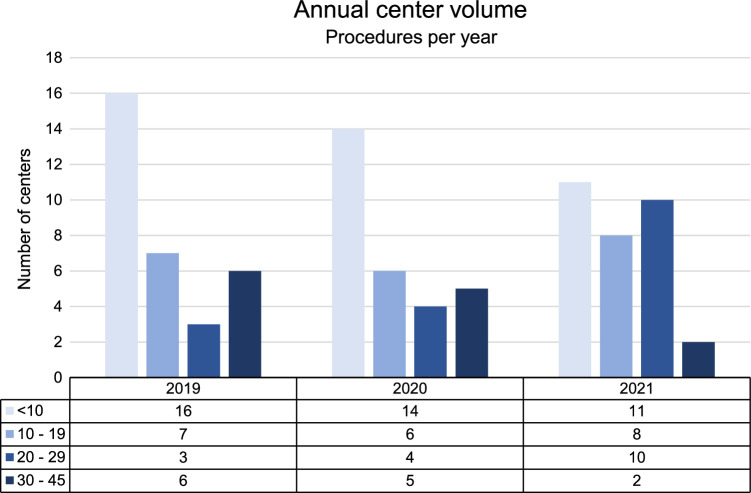
Annual center volume of minimally invasive pancreatoduodenectomy among participating centers during the study period.

Figure 1 shows the annual MIPD center volume among participating centers during the study period. Overall, the median annual volume of MIPD per center was 13 (IQR 8–46). Among all 45 centers performing MIPD, 20 performed R-MIPD, 15 performed L-MIPD, and 10 centers performed both. Over time, the median annual MIPD volume per center increased from 10 (IQR 4–22) in 2019 to 15 (IQR 7–24) in 2021 (*P*=0.462). The percentage of centers performing L-MIPD decreased from 46.9% (2019) to 25% (2021), while the number of centers performing R-MIPD increased from 46.9% (2019) to 65.6% (2021). See Table 1 for more details.

**Table 1:** Characteristics of centers performing MIPD in E-MIPS registry (2019–2021).

Center	Number (%)
Centers performing MIPD, *n* (%)	45 (62.5)
Annual volume MIPD, median (IQR)	13 (8–46)
Centers with annual volume ≥20, *n* (%)	
2019	9 (28.1), incl 6 R-MIPD
2020	9 (31), incl 4 R-MIPD
2021	12 (37.5) incl 8 R-MIPD
Centers performing R-MIPD, *n* (%)	
2019	15 (46.9)
2020	15 (51.7)
2021	21 (65.6)
Annual volume R-MIPD, median (IQR)	12 (6–45)
Centers performing L-MIPD, *n* (%)	
2019	15 (46.9)
2020	10 (34.5)
2021	8 (25)
Annual volume L-MIPD, median (IQR)	6 (2–25)
Centers performing both R-MIPD and L-MIPD, I (%)	
2019	2 (6.2)
2020	4 (13.8)
2021	3 (9.4)

IQR, interquartile range; L-MIPD, laparoscopic pancreatoduodenectomy; R-MIPD, robotic pancreatoduodenectomy.

From 21 centers, answers were collected regarding center-specific experience in MIPD, of which eight performed R-MIPD, seven performed L-MIPD, and six switched from L-MIPD (of which five centers during the study period, and one center before the studied period).

### Patient characteristics

In the total MIPD cohort, 48.1% of the 1336 patients were female (*n*=642), median age was 68 years (IQR 59–75), 64.8% of patients were ASA 1 or 2 (*n*=840), and 28.5% had previous abdominal surgery (*n*=335). In total, 12.5% had a BMI >30 kg/m^2^ and 63.9% of patients had a dilated pancreatic duct (>5mm). See Table 2 for more details.

**Table 2:** Baseline, intraoperative, and postoperative outcomes after MIPD.

	Overall MIPD	R-MIPD	L-MIPD	
Variable	*N*=1336	*N*=835	*N*=501	*P* ^#^
Patient characteristics				
Age, years, median [IQR]	68 [59–75]	69 [61–76]	66 [57–74]	**<0.001**
Sex, female, *n* (%)	642 (48.1)	394 (47.2)	248 (49.5)	0.445
BMI, median [IQR]	25 [22.4–27.8]	24.8 [22.5–27.6]	25.4 [22.3–28.2]	0.278
ASA 1–2, *n* (%)	840 (64.8)	486 (60.7)	354 (71.5)	**<0.001**
Comorbidity, *n* (%)	800 (60.2)	476 (57.4)	324 (64.7)	**0.010**
Previous abdominal surgery, *n* (%)	335 (28.5)	167 (24.3)	168 (34.4)	**<0.001**
Minimally invasive	146 (12.4)	77 (11.2)	69 (14.1)	0.162
Open	205 (17.4)	98 (14.3)	107 (21.9)	**0.001**
Vascular involvement, *n* (%)	120 (9.1)	80 (9.8)	40 (8.0)	0.322
Organ involvement, *n* (%)	92 (6.9)	59 (7.1)	33 (6.6)	0.817
Preoperative dilated duct >5 mm, *n* (%)	588 (63.9)	352 (65.7)	234 (61.3)	0.186
Neoadjuvant therapy, *n* (%)	92 (6.9)	54 (6.9)	38 (8.8)	0.273
Intraoperative outcomes				
Operative time, min, median [IQR]	404 [332–495]	410 [345–492]	390 [309–504]	**0.004**
Blood loss, mL, median [IQR]	200 [100–350]	200 [100–400]	200 [100–300]	0.407
Conversion, *n* (%)	129 (9.7)	81 (9.7)	48 (9.6)	1.000
Bleeding	21 (1.6)	11 (1.3)	10 (1.2)	0.460
Tumor advancement	11 (0.8)	6 (0.7)	5 (1.0)	0.815
Vascular involvement	29 (2.2)	13 (1.6)	16 (3.2)	0.073
Insufficient overview	16 (1.2)	11 (1.3)	5 (1)	0.795
Adhesions	17 (1.3)	13 (1.6)	4 (0.8)	0.344
Technical reason	23 (1.7)	8 (1)	15 (3)	**0.011**
Other	12 (0.9)	10 (1.2)	2 (0.4)	0.231
Drain placement, *n* (%)	1223 (92.8)	742 (90.8)	481 (96.0)	**<0.001**
Postoperative outcomes				
Major morbidity (CD grade ≥3), *n* (%)	551 (41.2)	380 (45.5)	171 (34.1)	**<0.001**
In-hospital/30-day mortality, *n* (%)	60 (4.5)	34 (4.1)	26 (5.2)	0.413
POPF grade B/C, *n* (%)	281 (22.7)	196 (25.4)	85 (18.2)	**0.004**
PPH grade B/C, *n* (%)	142 (10.8)	101 (12.4)	41 (8.2)	**0.023**
DGE grade B/C, *n* (%)	198 (15.4)	160 (19.4)	38 (8.3)	**<0.001**
Bile leakage grade B/C, *n* (%)	101 (7.7)	70 (8.6)	31 (6.2)	0.151
Percutaneous drainage, *n* (%)	333 (25.4)	247 (30.4)	86 (17.2)	**<0.001**
Reoperation, *n* (%)	144 (11.7)	94 (12.7)	50 (10.1)	0.192
Wound infection, *n* (%)	32 (2.4)	22 (2.7)	10 (2.0)	0.542
Length of stay, days, median [IQR]	12 [8–21]	12 [8–21]	12 [8–21]	0.827
Readmission, *n* (%)	187 (15.3)	135 (17.0)	52 (12.3)	**0.037**

Values in parentheses are percentages unless mentioned otherwise. Percentages may not add up due to rounding and missing data.

ASA, American Society of Anesthesiology; CD, Clavien–Dindo; IQR, interquartile range; L-MIPD, laparoscopic pancreatoduodenectomy; MIPD, minimally invasive pancreatoduodenectomy; R-MIPD, robotic pancreatoduodenectomy.

# *P*-value between R-MIPD and L-MIPD.

### Intraoperative outcome

The median operative time was 404 min (IQR 332–495) with a median estimated blood loss of 200 ml (IQR 100–350). The overall conversion rate in MIPD was 9.7%. No differences in conversion rate and estimated blood loss were found between R-MIPD and L-MIPD. Operative time was longer after R-MIPD compared to L-MIPD [410 min (IQR 345–492) vs. 390 min (IQR 309–504); *P*=0.004]. See Table 2 for all intraoperative outcomes.

### Postoperative outcome

Postoperative outcomes are presented in Table 2. Overall, the rate of major morbidity after MIPD was 41.2% (*n*=551) and the rate of in-hospital/30-day mortality 4.5% (*n*=60). With respect to the ISGPS defined complications (grade B/C), the rates of POPF were 22.7% (*n*=281), PPH 10.8%, bile leakage 7.7%, and delayed gastric emptying 15.4%. Overall, 11.7% of patients underwent a reoperation (*n*=144) and median length of hospital stay was 12 days (IQR 8–21). Comparing postoperative outcomes of R-MIPD and L-MIPD, significantly higher rates of major morbidity (45.5 vs. 34.1%, *P*<0.001), POPF grade B/C (25.4 vs. 18.2%, *P*=0.004), and PPH grade B/C (12.4 vs. 8.2%, *P*=0.023) were found after R-MIPD as compared to L-MIPD. Histological and oncological outcomes are presented in Table 3.

**Table 3:** Histological and oncological outcomes after MIPD.

	Overall MIPD	R-MIPD	L-MIPD	
Variable	*N*=1336	*N*=835	*N*=501	*P* ^#^
Histological and oncological outcomes				
Malignant disease *n* (%)	738 (64.1)	470 (59.6)	268 (73.6)	**<0.001**
Histological outcomes				**<0.001**
Adenoma	139 (11.2)	119 (14.6)	20 (4.6)	
Pancreatic ductal adenocarcinoma (PDAC)	593 (47.7)	385 (47.4)	208 (48.3)	
Neuroendocrine tumor (NET)	73 (5.9)	46 (5.7)	27 (6.3)	
Intraductal papillary mucinous neoplasm (IPMN)	128 (10.3)	97 (11.9)	31 (7.2)	
Mucinous cystic neoplasm (MCN)	8 (0.6)	6 (0.7)	2 (0.5)	
Solid-pseudopapillary neoplasm (SPN)	14 (1.1)	8 (1.0)	6 (1.4)	
Serous cystadenoma	13 (1.0)	8 (1.0)	5 (1.2)	
Chronic pancreatitis	28 (2.3)	20 (2.5)	8 (1.9)	
Ampullary carcinoma	129 (10.4)	55 (6.8)	74 (17.2)	
Other	68 (5.5)	48 (5.9)	20 (4.6)	
Cholangiocarcinoma	51 (4.1)	21 (2.6)	30 (7.0)	
Total lymph nodes median [IQR]	16 [12–23]	16 [12–22]	17 [13–23]	0.213
Involved lymph nodes median [IQR]	1 [0–4]	2 [0–4]	1 [0–2]	**<0.001**
R0-resection, *n* (%)	543 (74.7)	293 (73.1)	250 (76.7)	0.303
Tumor size, mm, median [IQR]	25 [15–32]	25 [17–35]	22 [11–30]	**<0.001**
Adjuvant therapy if indicated^*^, *n* (%)	383 (58.8)	278 (38.1)	172 (52.3)	**<0.001**

Bold values indicate statistical significance (*P*<0.05), Values in parentheses are percentages unless mentioned otherwise.

IQR inter quartile range.

* in case of malignancy.

# *P*-value between R-MIPD and L-MIPD. R0, microscopic radical resection (>1 mm).

### Surgical volume

The number of centers meeting the Miami Guidelines annual volume cut-off of ≥20 MIPDs increased from 9 (28.1%) in 2019 to 12 (37.5%) in 2021 (*P*=0.424). In 2019, 9 of the contributing 33 centers were high-volume centers, of which 6 performed R-MIPD and 3 performed L-MIPD. In 2021, 12 of the 32 centers were high-volume centers, of which 8 were R-MIPD centers, 3 L-MIPD centers and 1 center performed both. In 2019, nine centers performed ≤5 MIPD procedures, of which two were R-MIPD centers. In 2021, seven centers performed ≤5 MIPD procedures, of which six were R-MIPD centers.

Outcomes between high-volume and low-volume centers, and R-MIPD versus L-MIPD, are presented in Tables 4 and 5. No differences in major morbidity and mortality were observed between low-volume and high-volume centers for both approaches. In R-MIPD, operative time and the reoperation rate were significantly lower in high-volume centers, compared to low-volume centers. The rate of percutaneous drainage was significantly higher in high-volume R-MIPD centers, compared to low-volume R-MIPD centers (35 vs. 19.1%; *P*<0.001). In L-MIPD, high-volume centers had a lower conversion rate, compared to low-volume centers (5.2 vs. 18.1%; *P*<0.001). In high-volume centers, operative time was significantly longer in L-MIPD as compared to R-MIPD [430 min (340–520) vs. 330 min (269–420); *P*<0.001].

**Table 4:** Intraoperative and postoperative outcomes after MIPD stratified for annual volume based on the Miami Guidelines volume cut-off.

	Annual volume MIPD ≥20	Annual volume MIPD <20	
Variable	*N*=925	*N*=411	*P*
Intraoperative outcomes			
Robotic approach, *n* (%)	595 (64.3)	240 (58.4)	0.045
Operative time, min, median [IQR]	409 [342–490]	390 [300–510]	0.105
Blood loss, mL median [IQR]	200 [100–350]	200 [100–400]	0.805
Conversion, *n* (%)	68 (7.4)	61 (14.8)	**<0.001**
Bleeding	12 (1.3)	9 (2.2)	0.331
Tumor advancement	9 (1)	2 (0.5)	0.562
Vascular involvement	7 (0.8)	22 (5.4)	**<0.001**
Insufficient overview	6 (0.6)	10 (2.4)	**0.013**
Adhesions	11 (1.2)	6 (1.5)	0.886
Technical reason	7 (0.8)	16 (3.9)	**<0.001**
Other	6 (0.6)	6 (1.5)	0.256
Drain placement, *n* (%)	827 (91.1)	401 (96.6)	**0.001**
Postoperative outcomes			
Major morbidity (CD grade ≥3), *n* (%)	380 (41.1)	171 (41.6)	0.905
Mortality, *n* (%)	38 (4.1)	22 (5.4)	0.384
POPF grade B/C, *n* (%)	208 (22.5)	73 (17.8)	**0.047**
PPH grade B/C, *n* (%)	93 (10.1)	49 (11.9)	0.348
DGE grade B/C, *n* (%)	135 (14.6)	63 (15.3)	0.953
Bile leakage grade B/C, *n* (%)	77 (8.3)	24 (5.8)	0.149
Percutaneous drainage, *n* (%)	261 (28.2)	72 (17.5)	**<0.001**
Reoperation, *n* (%)	82 (8.9)	62 (15.1)	**0.001**
Wound infection, *n* (%)	22 (2.4)	10 (2.4)	1.000
Length of stay, days, median [IQR]	12 [8–21]	13 [9–20]	0.433
Readmission, *n* (%)	127 (13.7)	60 (14.6)	1.000

Values in parentheses are percentages unless mentioned otherwise. Percentages may not add up due to rounding and missing data.

ASA, American Society of Anesthesiology; CD, Clavien–Dindo; DGE, delayed gastric emptying; IQR interquartile range; POPF, postoperative pancreatic fistula; PPH, postpancreatectomy hemorrhage.

**Table 5:** Outcomes after R-MIPD and L-MIPD stratified for annual volume based on the Miami Guidelines volume cut-off.

	R-MIPD			L-MIPD		
Variable	Annual volume ≥20 *N*=595	Annual volume <20 *N*=240	*P*	Annual volume ≥20 *N*=330	Annual volume <20 *N*=171	*P*
Operative time, min, median [IQR]	402 [345–471]	450 [351–564]	**<0.001**	430 [340–520]	330 [269–420]	**<0.001**
Operative blood loss, mL median [IQR]	200 [100–400]	200 [100–400]	0.073	200 [100–300]	150 [100–300]	0.051
Conversion, *n* (%)	51 (8.6)	30 (12.5)	0.108	17 (5.2)	31 (18.1)	**<0.001**
Major morbidity (CD grade ≥3), *n* (%)	274 (46.1)	106 (44.2)	0.676	106 (32.1)	65 (38.0)	0.223
Mortality, *n* (%)	21 (3.5)	13 (5.4)	0.291	17 (5.2)	9 (5.3)	1.000
POPF grade B/C, *n* (%)	154 (27.7)	42 (19.4)	**0.021**	54 (17.9)	31 (18.6)	0.966
PPH grade B/C, *n* (%)	66 (11.4)	35 (14.9)	0.204	27 (8.2)	14 (8.3)	1.000
DGE grade B/C, n(%)	117 (19.7)	43 (18.5)	0.749	18 (6.2)	20 (11.8)	0.059
Bile leakage grade B/C, *n* (%)	56 (9.6)	14 (6.0)	0.121	21 (6.4)	10 (6.0)	1.000
Percutaneous drainage, *n* (%)	202 (35.0)	45 (19.1)	**<0.001**	59 (17.9)	27 (15.9)	0.663
Reoperation, *n* (%)	52 (9.8)	42 (19.9)	**<0.001**	30 (9.2)	20 (11.9)	0.425
Wound infection, *n* (%)	16 (2.8)	6 (2.5)	1.000	6 (1.8)	4 (2.3)	0.953
Length of stay, days, median [IQR]	12 [8–21]	12 [8–21]	0.335	12 [8–22]	13 [9–17]	0.979
Readmission, *n* (%)	99 (17.3)	36 (16.0)	0.728	28 (10.9)	24 (14.5)	0.341

Values in parentheses are percentages unless mentioned otherwise. Percentages may not add up due to rounding and missing data.

CD, Clavien–Dindo; DGE, delayed gastric emptying; IQR, interquartile range; POPF, postoperative pancreatic fistula; PPH, postpancreatectomy hemorrhage.

## Discussion

This international multicenter audit-based analysis including over 1300 patients undergoing MIPD in the E-MIPS registry found acceptable rates of major morbidity and in-hospital/30-day mortality across 45 centers in 14 European countries. Large differences were seen in annual MIPD volume between the participating centers, with only a third of the centers meeting the Miami volume cut-off of ≥20 MIPDs per year. Some differences were noted between low- and high-volume centers and between R-MIPD versus L-MIPD (i.e. for instance, in operative time, conversion, POPF grade B/C, and the use of postoperative percutaneous drainage.

In our study, the majority of centers performed R-MIPD (65.6% in 2021), which is in line with previous single-center and multicenter studies showing an increasing trend in the adoption of robotic surgery for pancreatic procedures^2,18–22^. This may reflect the increasing availability and adoption of robotic technology, but it will also be partly caused by the conflicting results of the LEOPARD-2 trial (2019), which have led to widespread concerns about the safety of L-MIPD. Despite this, the annual MIPD volume per center in our series remained relatively low, with a median of 15 procedures in 2021, which is a matter of concern. Although the number of centers meeting the Miami guidelines volume cut-off increased over time, only a minority of centers met the criterion in the study time period^8^.

Our analysis showed that both R-MIPD and L-MIPD had similar conversion rates and estimated intraoperative blood loss, but a longer operative time was found for R-MIPD. A recent systematic review and meta-analysis showed similar operative times between R-MIPD and L-MIPD^22^. The finding that more R-MIPD procedures were performed during the learning curve (2019–2021), as opposed to L-MIPD cases, may also have influenced these results. Moreover, centers that performed ‘both’ R-MIPD and L-MIPD during the study period, were mostly the centers who switched from L-MIPD to R-MIPD (6/10 centers). Thus, in these centers, approaches were not performed alternately during the same period. Only 4/10 centers performed both R-MIPD and L-MIPD alternately, because of this small population, no true comparison between centers who perform both R-MIPD and L-MIPD or centers who perform only R-MIPD or L-MIPD can be made in the current study. MIPS in general, either robotic or laparoscopic, performed by experienced surgeons in high-volume centers, might influence outcomes positively. However, the heterogeneity in annual and total MIPD volume of the participating centers and the current shift from laparoscopic to robot-assisted hampers a proper comparison between R-MIPD and L-MIPD regarding (intraoperative) outcomes, since surgeons are not equivalently trained. Therefore, this data may not be sufficiently mature for a direct comparison between R-MIPD and L-MIPD, since results appeared to be influenced also by the varying levels of expertise of the centers, rather than solely by the surgical approach.

Both the robotic and laparoscopic approach may different advantages during surgery. Namely, the robotic approach can offer more precise and easier suturing during the reconstruction phase, while the laparoscopic approach could offer more agility in moving between different quadrants during the resection phase. Moreover, variations in the robotic approach regarding the use of laparoscopic assistance and tools during the reconstruction phase have been described. In the current study, no evident advantages of R-MIPD over L-MIPD were observed. The true size of the impact of the current shift from R-MIPD to L-MIPD will have to be studied once more data are available in the E-MIPS registry besides that learning curves have been passed. The ideal surgical approach to MIPD could also be a combination between R-MIPD and L-MIPD based on the steps where each is most effective^23^.

In contrast to L-MIPD, the rate of POPF grade B/C differed significantly in R-MIPD between low-volume and high-volume centers, where similar major morbidity and mortality rates were found. Rates of POPF grade B/C after R-MIPD were actually higher in high-volume centers. This could be partly explained by the nationwide Dutch PORSCH trial as the majority of the high-volume R-MIPD cohort came from Dutch centers. PORSCH ran in all Dutch centers during the study period^24^ and implemented a multilevel algorithm for the early detection and treatment of POPF. This algorithm stimulated the liberal use of percutaneous catheter drainage for POPF and hereby actually reduced postoperative mortality on a nationwide level by 50%. This could explain the higher rate of major morbidity after R-MIPD in this cohort.

Overall, this international audit-based study reported a higher (9.7%) conversion rate as in previous MIPD series from expert centers. The Pittsburgh group found a 5.2% conversion rate among the first 500 R-MIPD at their center and the Dutch multicenter R-MIPD LAELAPS-3 training program, found a 6.5% conversion rate^2,19^. For L-MIPD, the conversion rate is in the range reported in the four published randomized trials on L-MIPD (ranging from 3 to 23.5%)^3,25–27^. Like in previous reports, this study confirms that a higher annual center volume reduced the conversion rate. Although this difference did not apply to R-MIPD, we found a significant difference in favor of the high-volume centers performing L-MIPD (5.2 vs. 18.1%; *P*<0.001). No difference was observed in the conversion rate between R-MIPD and L-MIPD, which is in contrast to earlier reports that found a lower conversion rate during R-MIPD versus L-MIPD^22,28^. This difference could be related to the fact that the surgeons in the laparoscopic group in the E-MIPS registry had more years of surgical experience.

Our study period (2019–2021), included the COVID-19 pandemic, which disrupted access to (oncological) surgical procedures in some countries. According to an Italian retrospective multicenter study, there was an overall significant reduction of all pancreatic resections performed, with a historical low-volume of 16 resections/month, compared with 43 average resections/month in 2019^29^. Clearly, the COVID-19 pandemic affected the annual volume and center volume during this study period, resulting in more low-volume centers.

The findings of this study should be interpreted in light of some limitations. First, the E-MIPS registry is not a mandatory audit. Therefore, we cannot guarantee the reproducibility of the results for the whole of Europe. Second, this study provides an overview of data from the first three years of the registry. Because of this short time period additional analyses, such as comparing low versus high-volume centers or changes in time, are limited. Third, with the emergence of robotic surgery, some centers have switched from laparoscopic to robotic surgery and therefore included R-MIPD cases from their first learning curve in this analysis. However, we have attempted to get an impression of the experience of the participating centers and surgeons in order to consider the learning curve of each center.

Fourth, the comparison of MIPD outcomes to open pancreatoduodenectomy outcomes was limited to the benchmark cut-offs and outcomes from the four published randomized trials on L-MIPD versus OPD. During the design of the E-MIPS registry, it was decided not to collect data on patients after OPD from the participating centers, as this would, by definition, introduce a considerable selection bias. Therefore, the results of the four currently unpublished randomized trials on MIPD versus OPD – the German EUROPA trial (DRKS00020407)^30^, the European DIPLOMA-2 trial (ISRCTN27483786), the Korean CORLAPPD (NCT03870698), and the Chinese PORTAL trial (NCT04400357)^31,32^ are awaited with great interest. Fifth, technical details on pancreatic anastomosis were not collected in the European registry. The main strength of this study is its large audit-based international multicenter design in an audit including a biannual audit visit of three randomly selected centers that is part of the protocol to ensure data validity. Furthermore, this audit-based design provides real world clinical data including a large sample size.

As the E-MIPS registry will continue to collect data in the upcoming years, an overview of the use and outcome of MIPS over a longer time period in Europe will follow in the future research.

## Conclusion

This is the first study to provide an overview of MIPD across Europe showing the feasibility of a large international prospective registry for MIPS including over 1000 patients annually. There were no differences observed in major morbidity and 30-day/in-hospital mortality between high-volume and low-volume centers. A large difference in annual MIPD volume between centers was found. High-volume centers were associated with lower rates of conversion rate and reoperation, while a higher rate of POPF grade B/C and postoperative drainage was found. There appears to be a strong shift toward R-MIPD; however, no evident advantages were observed in the current study, while comparing R-MIPD and L-MIPD outcomes. These findings are interesting whether we should ensure the current shift from L-MIPD to R-MIPD. However, variations in outcomes should be further evaluated by collecting data over a longer time period, when R-MIPD centers have passed the learning curve. The E-MIPS registry will continue to provide an overview of the use and outcome of MIPS in Europe aiming to safely implement MIPS.

## Ethical approval

Ethical approval was waived due to the observational nature of the study.

## Consent

Not applicable.

## Sources of funding

M.A.H. and M.G.B. received funding from Ethicon and Intuitive Surgical for the E-MIPS registry. The other authors have nothing to disclose relevant to this study or manuscript.

## Author contribution

A.M.L.H.E. and N.d.G.: drafted the manuscript; I.E.K., O.R.B., S.D., U.B., B.G.K., G.F., I.Q.M., O.S.-M., M.R., D.J.L., J.S.D.M., M.D.P.L., T.K., M.D’.H., F.R.S., B.E., T.H., M.S.L.L., A.I.-K., H.C.V.S., M.M., R.F.D.W., E.F.K., B.A., S.F., R.I., P.T., M.G.B., M.A.H.: participated in the design of the study and interpretation of the data during several meetings; A.E. and N.d.G.: performed and/or cross-checked the statistical analyses. All authors critically reviewed the manuscript, approved the final version, and are fully aware of this publication.

## Conflicts of interest disclosure

M.A.H. and M.G.B. received funding from Ethicon and Intuitive Surgical for the E-MIPS registry. N.d.G., M.A.H., and M.G.B. received funding from Intuitive Surgical for the international DIPLOMA-2 randomized trial on minimally invasive and open pancreatoduodenectomy in Europe. M.G.B., I.Q.M., B.G.K., and D.J.L. are involved as proctor for Intuitive Surgical for the LEARNBOT study on implementation and training of robot pancreatoduodenectomy in Europe. The other authors have nothing to disclose relevant to this study or manuscript.

## Research registration unique identifying number (UIN)

Study was registered at clinicaltrials.gov, UIN: NCT06135233, record status: public. clinicaltrial.gov/study/NCT06135233

## Guarantor

Professor M. Abu Hilal and Professor M.G. Besselink.

## Data availability statement

Datasets generated during and/or analyzed during the current study are available upon reasonable request.

## Provenance and peer review

Not applicable.
